# First isolation, *in-vivo* and genomic characterization of zoonotic variegated squirrel Bornavirus 1 (VSBV-1) isolates

**DOI:** 10.1080/22221751.2020.1847604

**Published:** 2020-11-20

**Authors:** Kore Schlottau, Daniel Nobach, Christiane Herden, Stefan Finke, Martin Beer, Donata Hoffmann

**Affiliations:** a Institute of Diagnostic Virology, Friedrich-Loeffler-Institut, Greifswald-Insel Riems, Germany; b Justus-Liebig-Universität, Institut für Veterinär-Pathologie, Gießen, Germany; c Center of Mind, Brain and Behavior, Justus-Liebig-University Gießen, Gießen, Germany; d Institute of Molecular Virology and Cell Biology, Friedrich-Loeffler-Institut, Greifswald-Insel Riems, Germany

**Keywords:** Variegated squirrel bornavirus 1, VSBV-1, virus isolation, animal trial, Lewis rats

## Abstract

The variegated squirrel bornavirus 1 (VSBV-1), a member of the family *Bornaviridae,* was discovered in 2015 in a series of lethal human infections. Screening approaches revealed kept exotic squirrels as the putative source of infection.

Infectious virus was successfully isolated by co-cultivation of infected primary squirrel cells with permanent cell lines. For *in vivo* characterization, neonatal and adult Lewis rats were inoculated either intracranially, intranasally or subcutaneously. After 4.5 months, three out of fifteen neonatal intracranially inoculated rats were VSBV-1 genome positive in the central nervous system without showing clinical signs. Pathohistological examination revealed a non-purulent encephalitis. While infection of immune incompetent rats (neonatal) using the type species of mammalian bornaviruses, the Borna disease virus 1, proceed to an immune tolerant status, VSBV-1 infection could result in inflammation of neuronal tissue. Sequencing showed minor adaptations within the VSBV-1 genome comparing to the viral genomes from infected squirrels, cell cultures or rat tissues.

In conclusion, we were able to generate the first VSBV-1 isolates and provide *in vivo* animal model data in Lewis rats revealing substantial differences between VSBV-1 and BoDV-1. Furthermore, the presented data are a precondition for insights into the transmission and pathogenesis of this novel zoonotic pathogen.

## Introduction

Variegated squirrel bornavirus 1 (VSBV-1) is one of the most recently discovered zoonotic viruses associated with fatal encephalitis in human patients [1–4]. Variegated squirrels (*Sciurus variegatoides*), red-tailed squirrels (*Sciurus granatensis*), Finlayson’s squirrels (*Calloscirurus finlaysonii*) and Swinhoei’s striped squirrels (*Tamiops swinhoei*) were tested positive for this pathogen, in addition to Prevost squirrels (*Callosciurus prevostii*), which exhibit even higher total case numbers [5,6]. While the squirrels positively tested for VSBV-1 genome appeared to be in good health, humans infected with VSBV-1 developed a severe and fatal immune-pathogenic mediated encephalitis [1–4].

VSBV-1 belongs to the genus *orthobornavirus* and is the exclusive member of the species *mammalian 2 orthobornavirus* up to now [7]. The enveloped viral particles consist of a non-segmented, negative sense, single stranded RNA genome [8]. Orthobornaviruses of mammals are recognized as neurotropic viruses inducing persistent infections. The type species of the family *Bornaviridae* is the Borna Disease virus 1 (BoDV-1), a well-known pathogen of horses and sheep in central Europe [9]. In addition, very recently also BoDV-1 was indisputably identified as causative agent of lethal human encephalitis cases [10–14]. Humans, sheep and horses are accidental hosts of BoDV-1 meaning that these species do not contribute to transmission of the agent in a population but suffer from a severe and fatal neurological disease (Borna disease) due to a severe immune-mediated non-purulent meningoencephalitis. Contrarily, infection of the bicolored white-toothed shrew (*Crocidura leucodon*) identified as reservoir host of BoDV-1 is characterized by an asymptomatic course despite widespread virus distribution and continuous virus shedding via various routes [15,16]. Therefore, the pathogen–host interactions of VSBV-1 in exotic squirrels display characteristics of reservoir host - virus interfaces (asymptomatic infection), while contrastingly shedding of VSBV-1 in excretions from squirrels was of minor load and cohabitant squirrels stayed non-infected [5,6]. For BoDV-1, it is known that the development of immune-mediated pathogenesis and disease in infected rodents is dependent on age and the immune status as well as on the genetic background of the host. For instance, severe immune-mediated disease occurs in adult immune competent Lewis rats whereas in contrast, infection of immune incompetent neonatal Lewis rats leads to lifelong persistence with only minor subclinical deficiencies. BoDV-1 is restricted to the central and peripheral nervous system in adults but can be detected in all tissues in neonatally infected Lewis rats [17–20].

*In vivo* animal infection data, like e.g. an experimental evidence for the immune-mediated pathogenesis of VSBV-1-induced encephalitis as described in human VSBV-1 cases [4], were missing due to a lack of virus isolates and hitherto existing *in vivo* infection models.

Here, we describe the isolation and subsequent genomic and *in vivo* characterization of VSBV-1 as basis for future experiments concerning the transmission and pathogenesis of this novel zoonotic agent.

## Materials and methods

### Virus isolation

Naturally VSBV-1 infected squirrels were euthanized due to disease control efforts [5]. Virus isolation by co-cultivation was successful from three *Callosciurus prevostii* originating from two zoos and one private breeder from Germany (described as zoological gardens K and L as well as breeder E in Tappe et al. [2]). Those animals were tested VSBV-1 positive by a surveillance approach. Kidney as well as brain of these squirrels were used to obtain primary cells by either three times trypsin treatment or in case of the brain, as well as an explant culture. The cells were maintained for four weeks in Ham’s F12/IMDM (1:1) medium supplemented with 10% FCS and 1% penicillin, streptomycin (Merck, Darmstadt, Germany) and kanamycin (Merck) at 37°C and 5% CO_2_. After that, primary cells were co-cultivated with Vero 76 (Collection of Cell Lines in Veterinary Medicine CCLV-RIE 228) or C6 (CCLV-RIE 1452) cells (1:6). The mixed cell cultures were passaged twice a week in medium containing antibiotics until primary kidney or brain cells became extinct as indicated by a morphological uniform cell culture. VSBV-1 RNA loads of the sequential passages were monitored by RT-qPCR until indirect immunofluorescence revealed 100% VSBV-1 persistently infected Vero and C6 cell cultures. The percentage of infected cells was continuously tested by indirect immunofluorescence as described for the preparation of virus stocks. All three virus isolates, named “Alvin”, “Simon” and “Theodore”, were generated using the same procedure.

### Preparation of VSBV-1 virus stocks

VSBV-1 virus stocks for the animal experiments were obtained from persistently infected Vero cells. Cells were collected by trypsinisation, washed with PBS and sonicated (Brandson, Sonifier 450, Emerson, St Louis, MO, USA) in MEM (H) + MEM (E) media. After centrifugation, the supernatants were frozen at −80°C until further use. Virus stocks were titrated on Vero cells in 96 well microtiter plates to calculate TCID_50_/ml. Five days after infection, the cells were fixed with 4% paraformaldehyde (PFA, Sigma-Aldrich, Taufkirchen, Germany) for 10 min, washed with PBS (Neofroxx, Einhausen, Germany) and permeabilized with 0.5% Triton-X-100 (Serva, Heidelberg, Germany) for 10 min. The fixed cells were incubated for 45 min with a VSBV-1 antibody positive (as well as a negative) squirrel serum (1:200, squirrel sera from another holding [6]) and afterwards incubated with a 1:500 diluted Alexa 488 conjugate goat anti-mouse IgG (FisherScientific, New Hamspire, USA). Virus stock titers were determined by fluorescent light microscopy according to the Reed & Muench method [21].

### Animal study design

In the initial animal experiment the first VSBV-1 isolate named “Alvin” was characterized in adult (group 1–3) as well as neonatal (group 4–6) Lewis rats (Charles River, Sulzfeld, Germany) by using three different inoculation routes (intracerebrally (i.c., group 1&4)), (intranasally (i.n., group 2&5) and subcutaneously (s.c., group 3&6)) with a group size of 15 animals each (Table 1). Per inoculation route, five-week old Lewis rats (nine females and six males for group 1–3), to study VSBV-1 in the immune competent animal, as well as three pregnant Lewis rats for the generation of newborn Lewis rats, were purchased. Neonatal Lewis rats (group 4–6), in total 15 from three different litters, were inoculated within 24 h after birth. All animals received 10 µl VSBV-1 “Alvin” virus suspension corresponding to 5 × 10^3^ tissue infectious dose 50 (TCID_50_). The animals were monitored daily for clinical signs and bodyweight was determined weekly. Oral swabs samples from each individual as well as fecal pool samples per cage unit were collected weekly to monitor for virus shedding. After 90 days, five animals per group were sacrificed. The remaining ten animals were monitored for another 50 days until they were euthanized and subjected to autopsy (Table 1).

**Table 1.** Study design and outcome of VSBV-1 infections experiments carried out in Lewis rats.

Virus isolate	group ID	Age of animals	Inoculation route	Number of animals	Study duration	Tissue VSBV-1 RT-qPCR positive	Bornavirus reactive antibodies	Histological changes related to bornaviruses
**Alvin**	1	adult	intracerebrally	15	90/140 days	0/15	0/15	0/15
	2	adult	intranasally	15	90/140 days	0/15	10/15	0/15
	3	adult	subcutanously	15	90/140 days	0/15	0/15	0/15
	4	neonatal	intracerebrally	15 (+3 mothers)	90/140 days	3/15	6/15 (+2/3)	3/15
	5	neonatal	intranasally	15 (+3 mothers)	90/140 days	0/15	1/15 (+1/3)	0/15
	6	neonatal	subcutaneously	15 (+3 mothers)	90/140 days	0/15	4/15 (+1/3)	0/15
**Simon**	D	neonatal	intracerebrally	10 (+1 mother)	112 days	4/10	4/10	n.d.
**Theodore**	E	neonatal	intracerebrally	4 (+1 mother)	112 days	2/4	2/4	n.d.

n.d.= = not done.

The other two VSBV-1 isolates “Simon” (group D) and “Theodore” (group E) were used to inoculate each one litter of one pregnant Lewis rat intracerebrally. Thereby, ten newborn rats were available for the animal study with VSBV-1 “Simon” and four animals for the study with VSBV-1 “Theodore”. Those animals were euthanized and sera collected after monitoring for 112 days (Table 1).

### RNA extraction and detection of VSBV-1 by RT-qPCR

Total RNA was extracted from cell culture as well as oral swabs, faecal pool samples, and tissue samples using the NucleoMagVet kit (MachereyNagel, Düren, Germany) according to the manufacturer’s instructions in an elution volume of 100 μL. VSBV-1 RNA was detected by quantitative RT–PCR using two different VSBV-1 specific mixes: Squirrel mix 6.1 (L gene; 600 nM BDV-like-3847-F (GTC TGT CCT CAA ACT CTA CTG A), 600 nM BDV-like-3971-R (GGA ACG ACC CTC TGT GAA ATC), 200 nM BDV-like-3903-FAM (FAM-TCG TCC AAC CGG CCG TCT ATC AGG-BHQ1)) or Squirrel mix 10.1. (P/X gene; 600 nM BDV-like-7671-F (GTG GTG TCT GGA GAC TCT C), 600 nM BDV-like-7765-R (CTC ACA CTG CTT GAA CTC ATC A), 200 nM BDV-like-7712-FAM (FAM-AGA CTC GAG GGG CGC GAT GCC AT-BHQ1)) in a total reaction volume of 12.5 µl using the qScript XLT one-step RT-qPCR ToughMix Kit (Quanta BioSciences, Gaithersburg, USA). The following thermal program was applied: 1 cycle of 50°C for 10 min and 95°C for 1 min, followed by 45 cycles of 95°C for 10 s, 57°C for 30 s, 68°C for 30 s. The VSBV-1 specific mixes were combined with an internal ß-actin control mix [22] to assess sample quality. A quantified VSBV-1 standard curve as well as non-template controls were included in each RT-qPCR run. All real-time RT-PCRs were performed with the CFX96 Real-Time PCR Detection System (Bio-Rad, Hercules, USA).

### Dideoxy chain-termination “Sanger” sequencing and sequence analysis

To obtain entire coding sequences including the N/X and M/G intergenic regions, dideoxy chain-termination sequencing was performed as described elsewhere [5]. The newly sequenced VSBV-1 genomes from the cell culture isolates as well as animal passages were compared to the three VSBV-1 sequences originating from the infected exotic squirrels (accession number LT594388 (BH 133/15, “Alvin”), KY508799 (DH 06/16, “Simon”) and KY508798 (DH 08/16, “Theodore”)) to check for nucleotide and amino acid variations by using MUSCLE alignment [23] with the Geneious Prime software (version 2019.2.3.) [24].

### Detection of bornavirus-reactive serum antibodies

Lewis rat sera were screened for the presence of bornavirus-reactive IgG by indirect immunofluorescence assays (iIFA) as described elsewhere [10,14]. Briefly, BoDV-1 He80 infected cells were mixed 1:5 with uninfected Vero cells and incubated overnight at 37°C and 5% CO_2_. Cells were fixated and permeabilized. Then, cells were incubated with a twofold dilution of rat sera (starting from 1:20) for 45 min. For visualization, cells were incubated with a 1:500 diluted Cy3 labeled goat-anti-rat-IgG antibody (Jackson Immunoresearch, Pennsylvannia, USA). Cells were analysed by fluorescent microscopy in a 96 well microtiter plate. Antibody titers were given as log_2_ from the reciprocal of the highest positive dilution. Wells with uninfected Vero cells as well as negative rat sera served as negative controls.

### Confocal microcopy

Persistently VSBV-1 infected Vero cells were fixed with 80% acetone, followed by rehydration and blocking with milk power. Cells were incubated for 2 h at 37°C with a bornavirus-reactive serum sample from a human patient (1:500) and mouse-anti-ß-tubulin (1:1000, Sigma Aldrich, Missouri, USA). Following subsequent washing with PBS, cells were incubated (1 h, 37°C) with anti-human-Alexa-488 (1:1000 in PBS, Fisher Scientific, New Hampshire, USA) and anti-mouse-Alexa-568 (1:1000 in PBS, Fisher Scientific, New Hampshire, USA) as secondary antibodies as well as Hoechst33342 (1 µg/ml) to make the nuclear chromatin visible. The specimens were mounted on coverslips and z-stack images (z-step size 0.35 µm) were acquired by confocal laser scanning microscopy (Leica DMI 6000 TCS SP5). Images were processed by using the Fiji, ImageJ (v1.52 h), distribution package [25,26].

### Pathology: necropsy, histopathology, immunohistochemistry and in situ hybridization

At necropsy, the following tissues were collected for RNA isolation and RT-qPCR: cerebellum, cerebrum, spinal cord, parotid salivary gland, lung, heart, liver, spleen, kidney, bladder, muscle (hind leg), skin (abdomen). Furthermore, the following tissues were used for fixation in 4% neutral-buffered formalin (Carl Roth, Karlsruhe): from all animals cerebellum and cerebrum and from at least five animals per group spinal cord, parotid salivary gland, lung, heart, liver, spleen, kidney, bladder, muscle, skin and nasal concha. Fixed tissues were embedded in paraffin, and 2 μm sections were stained with hematoxylin and eosin for light microscopical examination.

For VSBV-1 antigen detection, immunohistochemistry (IHC) with polyclonal cross-reactive bornavirus-specific antibody p24 was performed on PCR-positive animals as described elsewhere [1]. Additionally, IHC was performed for one PCR-negative animal per group. Shortly, deparaffinized, H_2_O_2_-treated slides were blocked with 5% goat serum and incubated with the polyclonal rabbit-antibody p24 (dilution 1:2000) overnight. Negative control slides were incubated with rabbit serum. As secondary antibody, goat-IgG against rabbit (Agilent, Santa Clara, CA, USA) was used and detected with avidin–biotin-complex with DAB as substrate. Antigen distribution and quantity was scored according to Petzold et al. [27].

Viral RNA distribution was further evaluated by in-situ hybridization with RNA-Scope probes according to manufacturer’s instructions, using RNAscope 2.5 HD Assay-BROWN (ACD, Advanced Cell Diagnostics, Newark, CA) and RNAscope probe V-VSBV1-N. The same score was applied for viral RNA distribution as for viral antigen [27].

### Ethics

The animal experiments were evaluated and approved by the ethics committee of the State Office of Agriculture, Food safety, and Fishery in Mecklenburg – Western Pomerania (LALLF M-V: LVL MV/TSD/7221.3-1.1-015/18 and 7221.3-2-010/18). All procedures were carried out in approved biosafety level 3 (BSL3) facilities.

## Results

### Virus isolation, propagation and cell culture adaption

**Figure 1.**
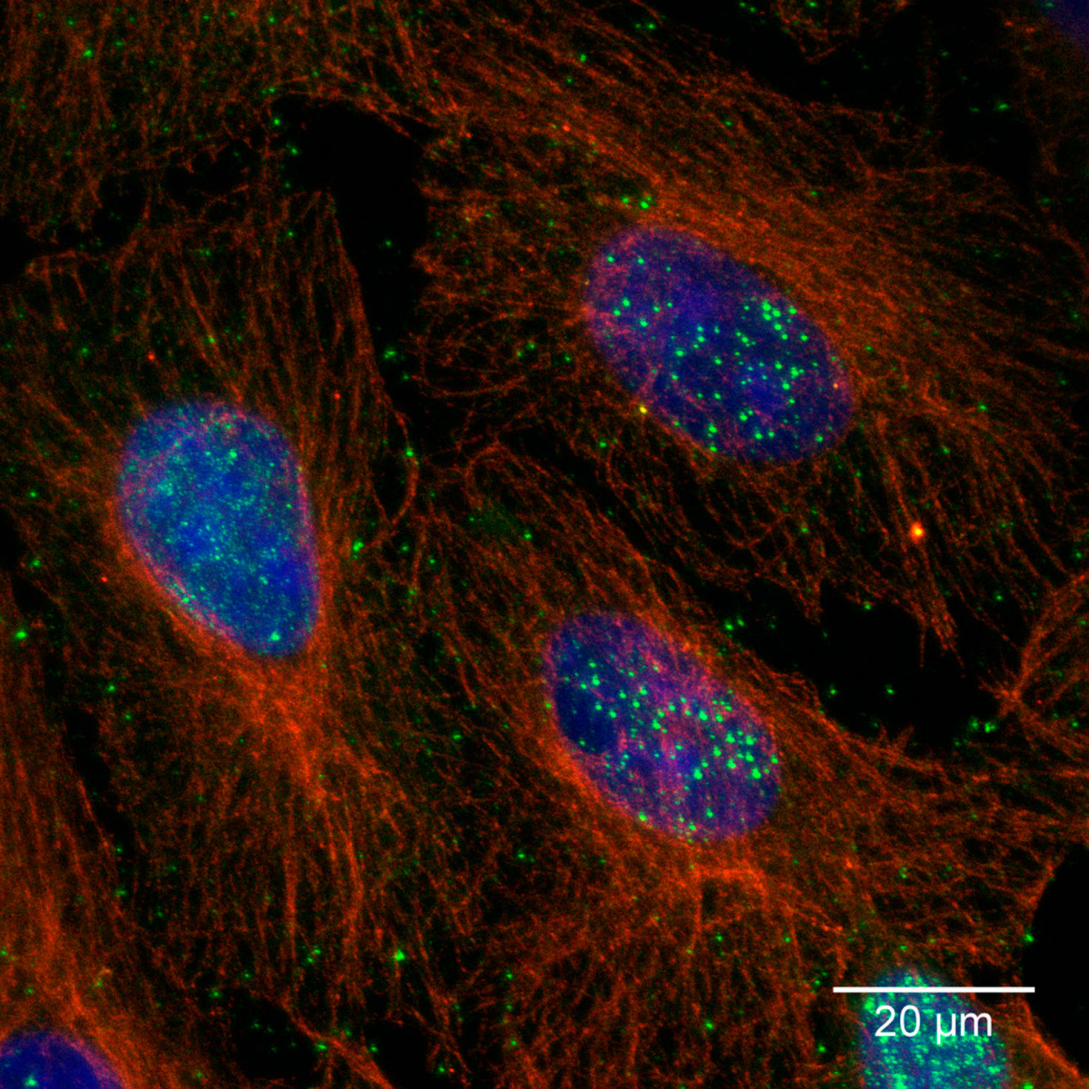
VSBV-1 isolate “Alvin” on Vero cells. Bornavirus specific nuclear and cytoplasmic dots (green); cytoplasmic microtubuli (red) and nuclear chromatin (blue). Maximum-z-projection of three confocal slices acquired at a z-step size of 0.35 µm.

In total, VSBV-1 virus isolation was successful from brain as well as kidney tissue from three *Callosciurus prevostii* by co-cultivation with established permanent cell cultures. While the primary cells became extinct within the co-culture, VSBV-1 replicated within Vero and C6 cells. Incubation of VSBV-1 infected cells with bornavirus-reactive antibody containing sera revealed nuclear accumulation of dots typical for bornaviruses in all cell cultures (Figure 1).

**Figure 2.**
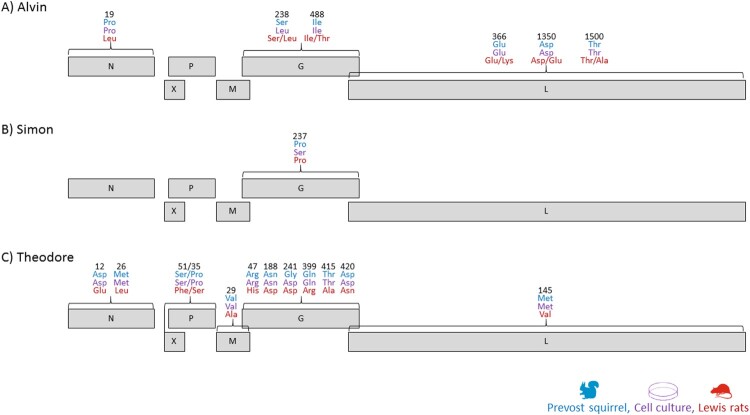
Schematic overview of the amino acid mutations during the adaptation from the squirrel to the cell culture and also to the Lewis rat passage. Given are the amino acid positions within the protein, respectively.

Sequencing revealed only minor changes in nucleotide and amino acid sequence leading to over 99.9% sequence similarity compared to the original squirrels based VSBV-1 sequence, respectively (Figure 2, Table S1). For the first VSBV-1 isolate “Alvin” one amino acid change occurred within the G protein (S238L) from the squirrel derived VSBV-1 sequence to the cell culture adapted VSBV-1 isolate. The other two isolates featured as well each one amino acid substitution within the G protein (“Simon” P237S and “Theodore” G241D) as a substitution during adaption to Vero cell culture. The persistently infected Vero cells were passaged multiple times, but at least up to passage no. 30 no further sequence changes occurred.

### VSBV-1 infection studies in Lewis rats

None of the inoculated Lewis rats showed any signs of disease or reduction of bodyweight, regardless of their age at the time of inoculation or the used infection route. The growth rates of the neonatally infected rats were within a physiological range. No viral shedding could be observed meaning that all oral swabs and faecal samples remained VSBV-1 genome negative for the whole study period for all three VSBV-1 isolates (90, 112 or 140dpi).

**Figure 3.**
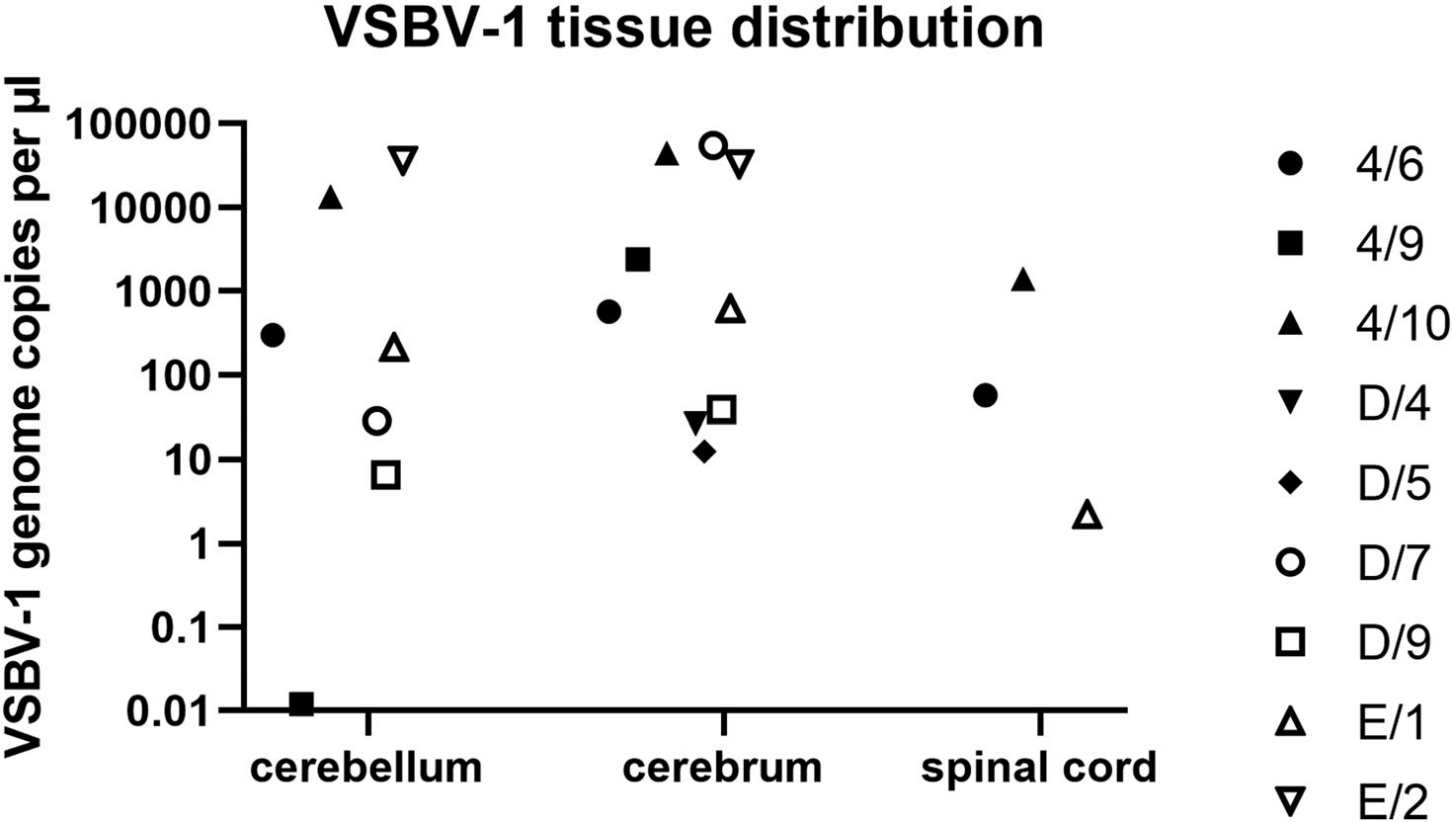
VSBV-1 genome load in different organs of positive animals. Salivary gland, lung, heart, liver, spleen, kidney, bladder, muscle and skin were negative.

The first infection experiment using VSBV-1 “Alvin” had the aim to (I) characterize VSBV-1 in adult as well as neonatal rats and (II) to analyse differences concerning the infection route. No viral genome was detected in any of the tissues from the 30 animals (five animals per group) tested after 90 days. All residual rats were euthanized after 140 days and tissues analysed by RT-qPCR. Thereby, VSBV-1 RNA could be detected in the central nervous system of three neonatally intracerebral infected Lewis rats (Group 4, #6, #9 and #10, Table 1). Those three animals had varying amounts of VSBV-1 RNA within the cerebrum (genome copies per µl template RNA between 4.4 × 10^4^ and 5.7 × 10^2^), cerebellum (genome copies per µl template RNA between 1.3 × 10^4^ and 1.2 × 10^−2^) and spinal cord (only positive in animals 4/#6 and 4/#10, genome copies per µl template RNA 5.7 × 10^1^ and 1.4 × 10^3^, respectively). All other tissue samples were genome-negative (Figure 3).

The three RT-qPCR positive Lewis rats (group 4, neonatal intracerebral) showed bornavirus-reactive antibody titers (between 5.6 and 11.6, log_2_). Furthermore, other inoculated Lewis rats scored as well serologically positive: intranasally infected Lewis rats (group 2), a few of the non-inoculated female Lewis rats used to generate the newborns and neonatally infected animals (three additional rats from group 4, one from group 5 and four from group 6) (Table 3).

**Figure 4.**
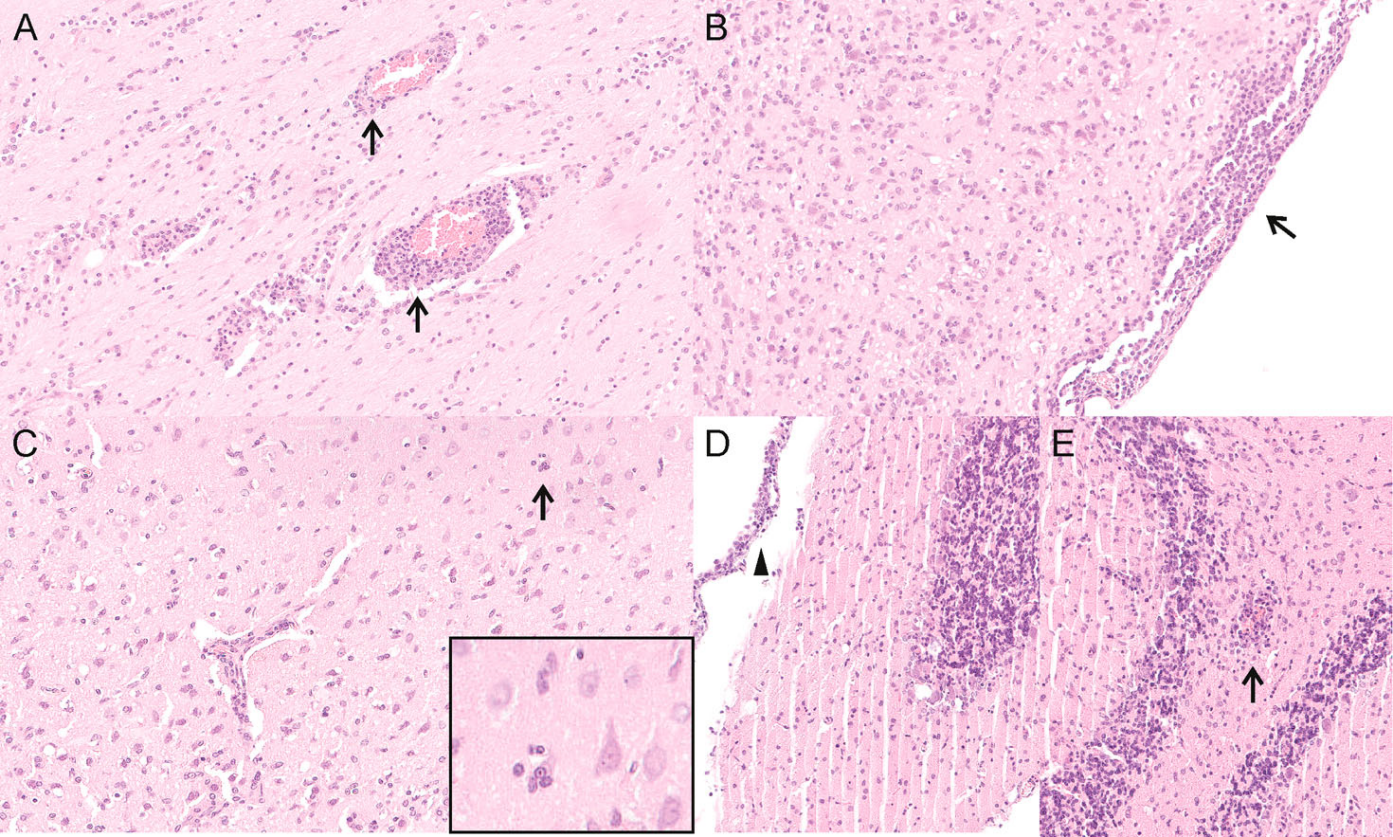
Histological lesions detected in neonatally intracerebral infected Lewis rats. (A) animal 4/#6, cerebral cortex, meningoencephalitis with mononuclear perivascular infiltrates (arrows). (B) animal 4/#6, brain stem, meningoencephalitis with mononuclear meningeal infiltrates (arrow). (C) animal 4/#6, cerebral cortex, meningoencephalitis with mononuclear perivascular infiltrates and satellitosis (arrow and insert). (D) animal 4/#10, cerebellum, meningoencephalitis with mononuclear meningeal infiltrates (arrowhead). (E) animal 4/#10, cerebellum, meningoencephalitis with mononuclear perivascular infiltrates (arrow).

Three animals (group 4, #6, #9, #10, neonatal intracerebral inoculation) displayed a moderate to severe non-purulent meningoencephalitis with mononuclear perivascular cuffs, multifocal lymphohistiocytic meningitis and activation of glia cells (Figure 4, Table 2). Five animals (group 4, animal #1, #2, #3, group 5, #3, #13) showed proliferation of meningeal cells and nearly all animals showed background lesions like congestion of the lung or follicular hyperplasia of the spleen (Table S2).

**Table 2.** Excerpt of the histological results and virus distribution from VSBV-1 “Alvin” infection studies in Lewis rats.

**Animal number**	**Organ**		**Histopathology**	**Immunohistochemistry**		**In situ hybridisation**	
				Score	Cells positive for VSBV-1 antigen	Score	Cells positive for VSBV-RNA
**Group 4 /#6**	Brain	Cerebral cortex	Non-purulent meningoencephalitis with mononuclear perivascular cuffs, meningeal infiltrates, glia activation and satellitosis	++	Neurons, pyramidal cells, astrocytes	+	Neurons, astrocytes
		Hippocampus		++++	Neurons, astrocytes	++	Neurons, astrocytes
		Cerebellum		-		-	
		Brain stem		+++(+)	Neurons, astrocytes	++	Neurons, astrocytes
	Spinal cord		No histological lesion	+(+)	Neurons, ganglia cells, astrocytes	-	
	Pancreas		No histological lesion	+++	Endocrine cells of langerhans islets	-	
**Group 4 /#9**	Brain	Cerebral cortex	Non-purulent meningoencephalitis with mononuclear perivascular cuffs, meningeal infiltrates and glia activation	+	Neurons, pyramidal cells, astrocytes	+	Neurons, astrocytes
		Hippocampus		+++	Neurons, astrocytes	++	Neurons, astrocytes
		Cerebellum		-		-	
		Brain stem		-		-	
	Spinal cord		No histological lesion	-		-	
	Pancreas		No histological lesion	++(+)	Endocrine cells of langerhans islets	-	
	Adrenal gland		No histological lesion	+++	Medullar and cortical endocrine cells	-	
**Group 4 /#10**	Brain	Cerebral cortex	Non-purulent meningoencephalitis with mononuclear perivascular cuffs, meningeal infiltrates and glia activation	+	Neurons, pyramidal cells, astrocytes	++	Neurons, astrocytes
		Hippocampus		+++	Neurons, astrocytes	++	Neurons, astrocytes
		Cerebellum		+++(+)	Purkinje cell, astrocytes	+	Neurons, astrocytes
		Brain stem		++	Neurons, astrocytes	+	Neurons, astrocytes
	Spinal cord		No histological lesion	-		-	
	Pancreas		No histological lesion	++(+)	Endocrine cells of langerhans islets	-	

++++ = very high no of positive cells, +++ = high no. of positive cells, ++ = moderate no. of positive cells, + = low number of positive cells, - = no positive cells (according to Petzold et al., 2019).

**Figure 5.**
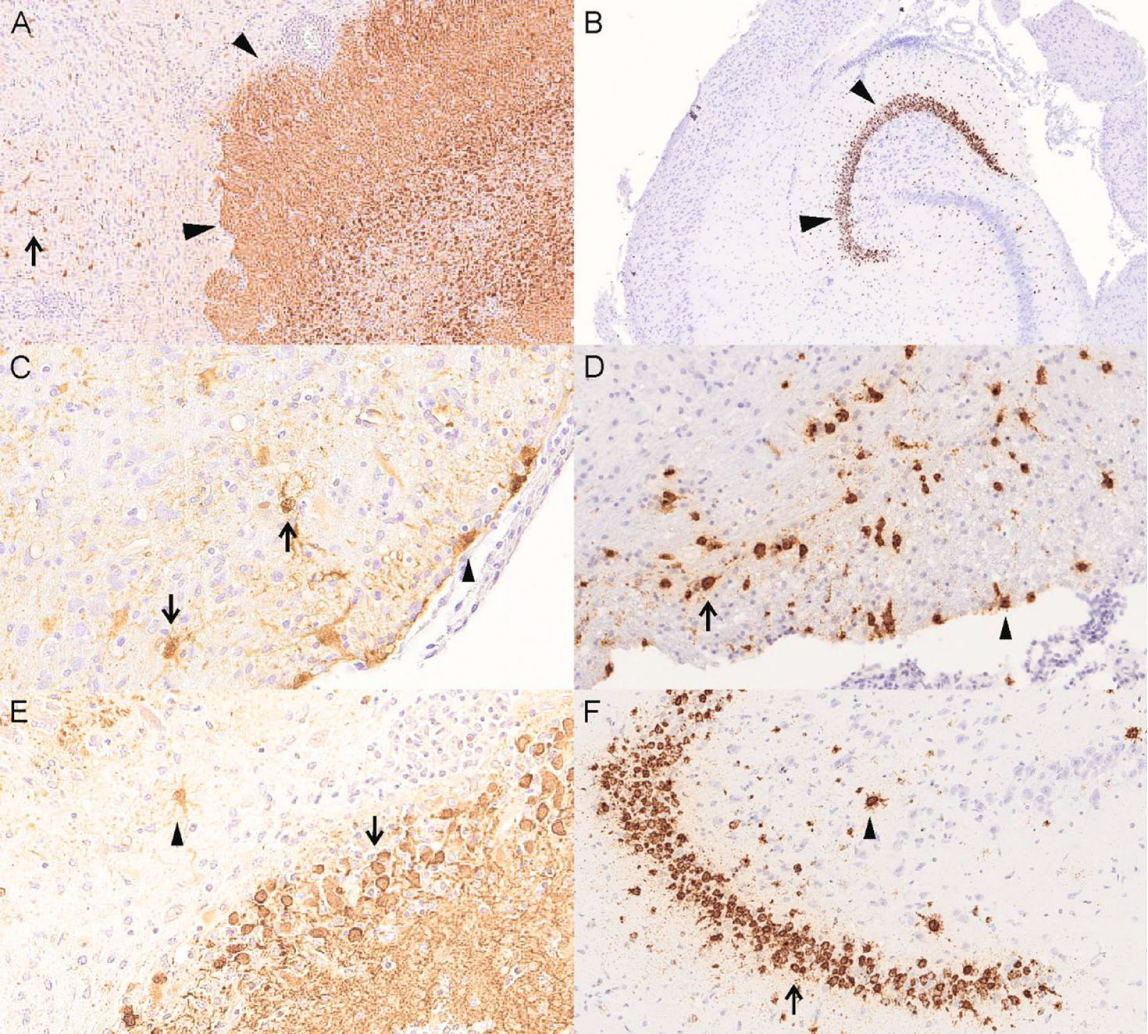
Viral distribution demonstrated by detection of viral antigen by immunohistochemistry (IHC) and viral genomic RNA by in-situ hybridization (RNAScope). (A) animal 4/#6, IHC, viral antigen is predominantly present in the hippocampus (arrowheads) and scattered in the cerebral cortex (arrows). (B) animal 4/#9, RNAscope, viral genomic RNA is predominantly present in the hippocampus. (C) animal 4#/6, IHC, brain stem, viral antigen present in neurons (arrows) and ependymal cells (arrowhead). (D) animal 4/#10, RNAscope, brainstem, viral genomic RNA is present in neurons (arrow) and ependymal cells (arrowhead). (E) animal 4/#6, IHC, hippocampus, viral antigen present in neurons (arrow) and astrocytes (arrowhead). (F) animal 4/#10, RNAscope, hippocampus, viral RNA present in neurons (arrow) and astrocytes (arrowhead).

Viral antigen was present in all three genome-positive animals (group 4, #6, #9, #10) predominantly in the hippocampus, but also in foci in the cerebral cortex and the brain stem (Figure 5, Table 2). In one animal (group 4, #6) very few virus antigen-positive cells were also present in the spinal cord, in another animal (group 4, #10) antigen was also present in the cerebellum. In all brain areas, antigen was found both intranuclearly and intracytoplasmatically in neurons, astrocytes and ependymal cells. Beside the nervous system, immunoreactivity was found in cells of neuroendocrine origin like cells of adrenal gland (group 4, #9) and the pancreatic islets (group 4, #6, #9, #10). All other tissues showed no immunoreaction.

By RNAScope, viral RNA was found in all three PCR-positive animals in neurons and astrocytes of the hippocampus and the cerebral cortex, two animals displayed viral RNA also in neurons and astrocytes of the brain stems and in animal 4/#10 viral RNA was also found in the cerebellum (Figure 5, Table 2). Adrenal gland and pancreatic islets as well as other organs displayed no viral RNA.

The other two VSBV-1 isolates were only used to inoculate i.c. newborn Lewis rats as a proof of principle. Again, not all animals could be infected, but four animals inoculated with VSBV-1 “Simon” (group D) and two animals inoculated with VSBV-1 “Theodore” (group E) had detectable amounts of VSBV-1 RNA (genome copies per µl template RNA between 5.5 × 10^4^ and 2.2 × 10^0^) within their central nervous systems without exhibiting clinical signs (Table 1, Figure 3). Most interestingly, animals successfully infected with VSBV-1 “Simon” or “Theodore” exhibited genome loads of similar value within the tested CNS tissues. VSBV-1 “Simon” and VSBV-1 “Theodore” infected animals displayed also bornavirus-reactive antibodies with titres between 5.6 and 7.6 (log_2_) (Table 3).

**Table 3.** Bornavirus-reactive antibody titers of VSBV-1 inoculated Lewis rats at the endpoint of the study. Exemplary serum samples taken before inoculation were below the cut off (< 1:20) for iIFA.

*animal number*	*Group 1*	*Group 2*	*Group 3*	*Group 4*	*Group 5*	*Group 6*	*Group D*	*Group E*	*Mothers of group 4*	*Mothers of group 5*	*Mothers of group 6*	*Mother of group D*	*Mother of group E*
*1*	-	9,6	-	-	-	-	-	5,6	-	-	-	-	-
*2*	-	8,6	-	-	-	-	-	7,6	5,6	5,6	-		
*3*	-	7,6	-	-	-	-	-	-	7,6	-	5,6		
*4*	-	-	-	-	-	5,6	6,6	-					
*5*	-	9,6	-	-	-	-	7,6						
*6*	-	-	-	10,6	-	7,6	-						
*7*	-	6,6	-	-	-	-	5,6						
*8*	-	-	-	-	-	7,6	-						
*9*	-	6,6	-	5,6	-	-	5,6						
*10*	-	7,6	-	11,6	-	-							
*11*	-	6,6	-	-	-	-							
*12*	-	-	-	7,6	6,6	6,6							
*13*	-	7,6	-	7,6	-	-							
*14*	-	5,6	-	7,6	-	-							
*15*	-	-	-	-	-	-							

- = below cutoff (first dilution 1:20), titers given as log_2_.

### Viral adaptations during Lewis rat passage

To check for adaptations within the VSBV-1 genome during animal passages, the VSBV-1 genomes from the three Lewis rats infected with VSBV-1 “Alvin”, four animals with VSBV-1 “Simon” and two animals with VSBV-1 “Theodore” were sequenced and compared to their cell culture derived inoculum sequence, respectively. The rat passage of VSBV-1 “Alvin” resulted in nucleotide changes at ten positions in total. Two of them occurred within the intergenic region between N and X and another two (one in the X gene and one on the G gene) did not lead to a change in the amino acid sequence. The other six nucleotide-changes led to amino acid substitutions at least in one of the three infected animals. Within the N protein one amino acid was changed in all three animals (P19L). The amino acid change at position S238L of the G protein, which occurred during cell culture adaptation, was lost in two of the three animals (4/#6 and 4/#10) and another mutation occurred in the same animals (I488T). The other three amino acid mutations within the L protein occurred only in a proportion of the animals (G366K, D1350E, T1500A). The amino acid adaptation within the G protein (P237S) of VSBV-1 “Simon” was lost after the rat passage. In addition, only three silent nucleotide exchanges appeared . The most mutations emerged in VSBV-1 “Theodore” after the rat passage. Here, in total 16 nucleotide changes resulted in ten further amino acid substitutions. Two within the N protein (D12E, M26L), one within the X and P protein (S51F and P35S), one within the M protein (V29A), five additional within the G protein (R47H, N188D, Q399R, T415A and D420N) and one within the L protein (M154A) (Figure 2, table S1).

## Discussion

For the first time, we were able to generate VSBV-1 isolates by a time-consuming co-cultivation approach consisting of a transient primary cell culture from infected squirrels. Thereby, we could show that VSBV-1 is genetically highly stable and conserved in persistently infected cell cultures. However, within all three VSBV-1 isolates at least one amino acid exchange occurred in the glycoprotein. The fact that these amino acid exchanges mostly mutated back after the Lewis rat passage at least for the VSBV-1 isolates “Alvin” and “Simon” argues very clearly for a necessary adaption process to allow efficient replication in cell cultures of other species origin. However, it could also be possible that some kind of viral quasi-species exist, which could not be detected by our sequencing approach. Mostly, the exchanged amino acids were conservative replacements. For BoDV-1, it is described that mutations in the glycoprotein as well as in the RNA polymerase can lead to variable clinical signs and pathological lesions [28]. Besides, mutations in the RNA polymerase as well as phosphoprotein were required to adapt BoDV-1 to mice and lead to an enhanced replication efficiency in Vero cells [29]. The here described three VSBV-1 isolates are a prerequisite to study the influence of distinct amino acid exchanges on the replication and the adaption to new hosts, and provide the opportunity to study pathogenesis as well as countermeasure approaches.

By infection of adult as well as neonatal Lewis rats with the VBSV-1 isolate “Alvin” by three different inoculation routes, we were able to demonstrate replicating virus only in three neonatally infected animals, which were inoculated by the artificial intracerebral route. Despite the quite long observation period (140 days), we did not see any signs of disease or viral shedding in these animals. Nevertheless, they showed a moderate to severe non-purulent meningoencephalitis accompanied by activation of glia cells. These lesions resemble the inflammatory pattern in the CNS found in humans infected with VSBV-1 [1,3,4]. Detailing the CNS lesions in human VSBV-1-infection led to the assumption of an immune-mediated pathogenesis with comparable but also different features as known for BoDV-1 infections [4]. Whether comparable immune mediated processes could be responsible for the inflammation in the CNS of VSBV-1 infected neonatal rats remains to be investigated, e.g. by studying the immune cell composition and virus specificity of T cells. However, this would be quite different to the well-studied BoDV-1 infection of neonatal Lewis rats which is characterized by a persistent infection with widespread virus distribution in nearly every organ accomplished by immune tolerance without severe inflammation in the CNS [17]. In contrast, BoDV-1 infection of adult Lewis rats leads to T cell-mediated encephalitis with similar lesions as in the observed neonatally VSBV-1 infected Lewis rats [18,19]. It should be noted that VSBV-1 infection (isolate “Alvin”) was only successful in 3 out of 15 neonatal rats inoculated intracerebrally and not by any other route or infection of adult animals probably due to low species adaptation. Thus, VSBV-1 infection of rat-adapted isolates could be helpful to address this question in further studies. The reason why and how VSBV-1 could trigger the immune system in neonatal Lewis rats instead of leading to immune tolerance also should to be the scope of future studies which are now possible with virus isolates from the first Lewis rat passages.

Whether a quicker virus replication and earlier recognition by the immune system in the important time phase for immune tolerance was functional in the rats with inflammatory lesions could be addressed by further kinetic infection studies. This hypothesis could be substantiated by the fact that the viral load detected by RT-qPCR in animal 4/#9 was lower compared to the animals 4/#6 and 4/#10. The animal 4/#9 had as well a lower level of inflammation and virus distribution was restricted to the cerebellum and small foci in the cerebral cortex, while the animals 4/#6 and 4/#10 had a higher level of inflammation and distribution involved more foci in the cerebral cortex, brain stem and cerebellum. The sequence generated from animal 4/#9 had a higher similarity to the cell culture derived inoculum than the sequences from the other two animals. Therefore, it could be speculated again that less adaption occurred in this animal leading to a lower replication rate. Notably, not merely those three RNA-positive animals developed bornavirus-specific antibodies, although they had the highest titers. Indeed, a few animals from the other groups, especially group 2 (adult, i.n. inoculated) displayed bornavirus specific antibodies. Generally, the antibody detection without genome detection could be due to an abortive infection. This has been described for healthy horses in Germany, where BoDV-1- specific serum antibodies are present rising significantly in endemic areas or stables with previous equine Borna disease cases [30]. Another possibility would be that only very few cells were infected e.g. in the olfactory epithelium or bulbus olfactorius, tissues which were not used for viral genome detection by RT-qPCR. The antibodies detected within the mother animals, which were not inoculated, arose probably from direct contact to the inoculum after infection of the newborns by cleaning and licking leading to intranasal and/or oral uptake [31].

For BoDV-1, depending on the used strain and inoculation route, the incubation period in adult Lewis rats is approximately three weeks and infected animals develop a fatal disease [19]. Neonatally infected Lewis rats become persistently infected without any ongoing associated cellular immune response in the brain. In those animals, the virus is not restricted to the central nervous system and shedding occurs by saliva and urine [17]. Interestingly, in contrast to Lewis rats, other rat species e.g. Wistar rats or black-hooded rats inoculated with BoDV-1 develop neither encephalitis nor clinical signs despite replication of the virus in the central nervous system [32]. In our VSBV-1 infection experiments, only a few neonatally inoculated animals were successfully infected without showing signs of infection despite a moderate to severe meningoencephalitis. It could be possible, that those animals would display signs of encephalitis at a later time point, meaning a much longer incubation period than described for BoDV-1 – maybe again due to a lack of species adaptation. Alternatively, the Lewis rat immune system would be able to interfere with VSBV-1 virus replication and limit infection as maybe indicated by the immune response of several animals, at least in the adult animals. Another option would be that VSBV-1 requires more adaption to this new host species, and serial passaging of the virus in experimentally infected Lewis rats would increase the infectivity and change the virulence as known for BoDV-1 [33]. These possibilities should be investigated in the future.

In summary, our study clearly showed that Lewis rats can be infected with VSBV-1, but only immune incompetent animals by artificial intracranial inoculation and with a very low overall infection rate. Therefore, these animals behave more like “dead-end hosts”, as the virus is restricted to the central nervous system, the encephalitis is similar to those observed in humans and no viral shedding occurred. Whether species adaptation of VSBV-1 lead to different outcomes has to be addressed in further studies. Hence, further efforts in establishing useful animal models are urgently needed, especially for testing potential treatment options that are currently not available.

## Conclusion

VSBV-1 and BoDV-1 are different. No small animal model is so far available for intervention or pathogenesis studies.

## Supplementary Material

Supplementary_material.docx
