# Supplementary material for: First isolation, in-vivo and genomic characterization of zoonotic variegated squirrel Bornavirus 1 (VSBV-1) isolates
Source: Emerg Microbes Infect. 2020 Nov 20;9(1):2474–84. doi: 10.1080/22221751.2020.1847604 (PMC7717607; doi:10.1080/22221751.2020.1847604)
Supplement: Supplementary_material.docx [file TEMI_A_1847604_SM6356.docx]

**Supplementary material**

Table S1: Nucleotide and amino acid variations in three VSBV-1 isolates (A = Alvin; B = Simon; C = Theodore). VSBV-1 was sequenced from samples of the original squirrel tissue, from whom a cell culture isolate was obtained and again after passage in rats.

| A) Alvin |  | Nucleotide | | | | | Amino Acid | | | | |
| --- | --- | --- | --- | --- | --- | --- | --- | --- | --- | --- | --- |
| Gene/ Protein | Position | Squirrel LT594388 | Vero cell culture | Rat 4/6 | Rat 4/9 | Rat 4/10 | Squirrel LT594388 | Vero cell culture | Rat 4/16 | Rat 4/9 | Rat 4/10 |
| N | 56 / 19 | C | C | T | T | T | Pro | Pro | Leu | Leu | Leu |
| N --> X | 1114 | C | C | T | C | T | - | - | - | - | - |
| N --> X | 1151 | C | C | C | T | C | - | - | - | - | - |
| X | 1210 / 13 | T | T | C | T | C | Ile | Ile | Ile | Ile | Ile |
| G | 2891 / 238 | C | T | C | T | C | Ser | Leu | Ser | Leu | Ser |
| G | 3243 / 355 | C | C | T | C | T | Thr | Thr | Thr | Thr | Thr |
| G | 3641 / 488 | T | T | C | T | C | Ile | Ile | Thr | Ile | Thr |
| L | 4731 / 366 | G | G | G | G | A | Glu | Glu | Glu | Glu | Lys |
| L | 7685 / 1350 | T | T | A | T | A | Asp | Asp | Glu | Asp | Glu |
| L | 8133 / 1500 | A | A | G | A | G | Thr | Thr | Ala | Thr | Ala |

| B) Simon |  | Nucleotide | | | | | | Amino Acid | | | | | |
| --- | --- | --- | --- | --- | --- | --- | --- | --- | --- | --- | --- | --- | --- |
| Gene/ Protein | Position | Squirrel KY508799 | Vero cell culture | Rat D/4 | Rat D/5 | Rat D/7 | Rat D/9 | Squirrel KY508799 | Vero cell culture | Rat D/4 | Rat D/5 | Rat D/7 | Rat D/9 |
| N | 426 / 142 | C | C | T | T | C | T | Ser | Ser | Ser | Ser | Ser | Ser |
| N | 984 / 328 | G | G | A | A | A | A | Gly | Gly | Gly | Gly | Gly | Gly |
| G | 2887 / 237 | C | T | C | C | C | C | Pro | Ser | Pro | Pro | Pro | Pro |
| L | 6248 / 871 | T | T | C | C | C | C | Val | Val | Val | Val | Val | Val |

| C) Theodore |  | Nucleotide | | | | Amino Acid | | | |
| --- | --- | --- | --- | --- | --- | --- | --- | --- | --- |
| Gene/ Protein | Position | Squirrel KY508798 | Vero cell culture | Rat E/1 | Rat E/2 | Squirrel KY508798 | Vero cell culture | Rat E/1 | Rat E/2 |
| N | 36 / 12 | C | C | A | A | Asp | Asp | Glu | Glu |
| N | 76 / 26 | A | A | C | C | Met | Met | Leu | Leu |
| N --> X | 1160 | T | T | C | C | - | - | - | - |
| X / P | 1328 / 51/35 | C | C | T | T | Ser/Pro | Ser/Pro | Phe/Ser | Phe/Ser |
| M | 1930 / 29 | T | T | C | C | Val | Val | Ala | Ala |
| G | 2318 / 47 | G | G | A | A | Arg | Arg | His | His |
| G | 2643 / 155 | T | T | C | C | Tyr | Tyr | Tyr | Tyr |
| G | 2740 / 188 | A | A | G | G | Asn | Asn | Asp | Asp |
| G | 2900 / 241 | G | A | A | A | Gly | Asp | Asp | Asp |
| G | 2919 / 247 | T | T | C | C | Pro | Pro | Pro | Pro |
| G | 3267 / 363 | C | C | T | T | Gly | Gly | Gly | Gly |
| G | 3374 / 399 | A | A | G | G | Gln | Gln | Arg | Arg |
| G | 3421 / 415 | A | A | G | G | Thr | Thr | Ala | Ala |
| G | 3436 / 420 | G | G | A | A | Asp | Asp | Asn | Asn |
| L | 4095 / 154 | A | A | G | G | Met | Met | Val | Val |
| L | 4433 / 266 | T | T | C | C | Cys | Cys | Cys | Cys |
| L | 6431 / 932 | G | G | A | A | Ser | Ser | Ser | Ser |

Table S2: Full pathological report of all animals.

| organ | histopathologic lesion | group and animal number |
| --- | --- | --- |
| brain | no pathologic lesion (only selected organ) | group 1 (#5,# 6, #7, #8, #9, #10, #11, #12); group 2 (#5, #6, #8, #9, #10, #11, #12), group 3 (#5,# 6, #7, #8, #9, #10, #11, #12), group 4 (#11, #12, #13, #14, #15); group 5 (#6, #8, #14), group 6 (#4, #5, #6, #7, #9, #10, #14, #15) |
|  | focal parenchymal hemorrhage | group 2 (#7) |
|  | multifocal meningeal proliferation | group 4 (#1, #2, #3, #5, |
|  | non-purulent meningoencephalitis with mononuclear perivascular cuffs | group 4 (#6, #9, #10) |
|  | meningeal infiltrates | group 4 (#6, #9, #10) |
|  | glia activation | group 4 (#6, #9, #10) |
|  | satellitosis | group 4 (#6) |
|  | meningeal proliferation | group 5 (#3, #7, #13) |
| spleen | congestion | group 1 (#1, #2, #3, #4, #13, #14, #15); group 2 (#1, #2, #3, #4, #13, #14, #15); group 3 (#1, #2, #3, #4, #13, #14, #15); group 4 (#1, #2, #3, #4, #5, #6, #7, #8, #9, #10, #M1, #M2, #M3); group 5 (#1, #2, #3, #5, #7, #9, #10, #12, #13, #15, #M1, #M2, #M3); group 6 (#1, #2, #3, #8, #11, #12, #13, #M1, #M2, #M3) |
|  | follicular hyperplasia | group 1 (#1, #2, #3, #4, #13, #14, #15); group 2 (#1, #2, #3, #4, #13, #14, #15); group 3 (#1, #2, #3, #4, #13, #14, #15); group 4 (#1, #2, #3, #4, #5, #6, #7, #8, #9, #10 #M1, #M2, #M3); group 5 (#1, #2, #3, #5, #7, #9, #10, #12, #13, #15, #M1, #M2, #M3); group 6 (#1, #2, #3, #8, #11, #12, #13, #M1, #M2, #M3) |
|  | extramedullary hematopoesis | group 1 (#2, #13, #14); group 2 (#1, #2, #3, #4, #13, #14, #15); group 3 (#1, #2, #3, #4, #13, #14, #15); group 4 (#1, #2, #3, #4, #5, #6, #7, #8, #9, #10, #M1, #M2, #M3); group 5 (#1, #2, #3, #5, #7, #9, #10, #12, #13, #15, #M1, #M2, #M3); group 6 (#1, #2, #3, #8, #11, #12, #13, #M1, #M2, #M3) |
| kidney | congestion | group 1 (#1, #2, #3, #4, #13, #14, #15); group 2 (#1, #2, #3, #4, #13, #14, #15); group 3 (#1, #2, #3, #4, #13, #14, #15); group 4 (#1, #2, #3, #4, #5, #6, #7, #8, #9, #10, #M1, #M2, #M3); group 5 (#1, #2, #3, #5, #7, #9, #10, #12, #13, #15, #M1, #M2, #M3); group 6 (#1, #2, #3, #8, #11, #12, #13, #M1, #M2, #M3) |
|  | multifocal hemorrhages | group 3 (#2, #13, #14, #15) |
| liver | extramedullary hematopoesis | group 1 (#1, #3, #13, #15), group 2 (#3, #4), group 3 (#1, #2, #3, #14); group 4 (#1, #2, #3, #5, #6, #7, #8, #9, #10, #M1, #M2, #M3); group 5 (#1, #2, #3, #5, #7, #9, #10, #12, #13, #15, #M1, #M2, #M3); group 6 (#1, #2, #3, #8, #11, #12, #13, #M2, #M3) |
|  | mild periportal inflammation | group 1 (#3, #4, #13); group 4 (#10, #M1, #M3), group 5 (#15, #M1, #M2), group 6 (#M1, #M2) |
|  | congestion | group 5 (#10) |
| lung | congestion | group 1 (#1, #2, #3, #4, #13, #14, #15); group 2 (#1, #2, #3, #4, #13, #14, #15); group 3 (#1, #2, #3, #4, #13, #14, #15); group 4 (#1, #2, #3, #4, #5, #6, #7, #8, #9, #10, #M1, #M2, #M3); group 5 (#1, #2, #3, #5, #7, #9, #10, #12, #13, #15, #M1, #M2, #M3); group 6 (#1, #2, #3, #8, #11, #12, #13, #M1, #M2, #M3) |
|  | BALT hyperplasia | group 1 (#1, #2, #3, #4, #14, #15); group 2 (#1, #2, #3, #4, #13, #14, #15); group 3 (#1, #2, #13, #14, #15); group 4 (#1, #2, #3, #4, #5, #6, #7, #8, #9, #10, #M1, #M2, #M3); group 5 (#1, #2, #3, #5, #7, #9, #10, #12, #13, #15, #M1, #M2, #M3); group 6 (#1, #2, #3, #8, #11, #12, #13) |
|  | multifocal alveolar hemorrhages | group 1 (#1, #2, #3, #4, #13, #14, #15); group 2 (#1, #2, #3, #4, #13, #14, #15); group 3 (#1, #2, #3, #4, #13, #14, #15); group 4 (#1, #2, #3, #4, #5, #6, #7, #8, #9, #10, #M1, #M2, #M3); group 5 (#1, #2, #3, #5, #7, #9, #10, #12, #13, #15, #M1, #M2, #M3); group 6 (#1, #2, #3, #8, #11, #12, #13, #M1, #M2, #M3) |
|  | alveolar edema | group 1 (#14), group 2 (#2, #3, #4, #14); group 3 (#2, #3, #4, #13, #14, #15); group 4 (#1, #2, #3, #4, #5, #6, #7, #8, #9, #10, #M1, #M2, #M3); group 5 (#1, #2, #3, #5, #7, #9, #10, #12, #13, #15, #M1, #M2, #M3); group 6 (#1, #2, #3, #8, #11, #12, #13, #M1, #M2, #M3) |
|  | focal granuloma with foamy cells | group 2 (#3); group 4 (#4) |
|  | emphysema | group 3 (#1, #2, #3, #14, #15); group 4 (#1, #2, #3, #4, #5, #6, #7, #8, #9, #10, #M1, #M2); group 5 (#1, #2, #3, #5, #7, #9, #10, #12, #13, #15); group 6 (#1, #2, #3, #8, #11, #12, #13) |
| nose | mild purulent rhinitis | group 1 (#4); group 2 (#3), group 3 (#3); group 4 (#6, #10), group 5 (#5) |
| mesenterial lymphnodes | follicular hyperplasia | group 1 (#4) |
| pancreas | mild focal mononuclear inflammation | group 1 (#15) |
